# Curriculum satisfaction and pre-service teachers’ social–emotional competencies: the serial mediation role of artificial intelligence anxiety and learning engagement

**DOI:** 10.3389/fpsyg.2026.1951569

**Published:** 2026-09-28

**Authors:** Asil Derin

**Affiliations:** Faculty of Education, Kafkas University, Kars, Türkiye

**Keywords:** artificial intelligence anxiety, curriculum satisfaction, learning engagement, serial mediation analysis, social–emotional competencies, teacher education

## Abstract

This study examined the serial mediation role of artificial intelligence anxiety and learning engagement in the relationship between pre-service teachers’ curriculum satisfaction and social–emotional competencies. This study included 550 pre-service teachers from a public university in Türkiye. This correlational study used the IBM SPSS PROCESS macro (Model 6-Two-Mediator Serial Mediation Analysis/Model). In this study, the scales of student satisfaction, artificial intelligence anxiety, student engagement, and social–emotional expertise were used as data collection tools. To provide a coherent and integrated understanding of the structural relationships, this study was grounded in the input-process-output framework. In this model, curriculum satisfaction was regarded as an input. Artificial intelligence anxiety and learning engagement were regarded as processes. Social–emotional competencies were regarded as an output within the framework. As a result, curriculum satisfaction was negatively associated with artificial intelligence anxiety. Artificial intelligence anxiety was negatively associated with learning engagement. Learning engagement was positively associated with social–emotional competencies. Curriculum satisfaction was also positively associated with social–emotional competencies. When the model included artificial intelligence anxiety and learning engagement simultaneously, the relationship between curriculum satisfaction and social–emotional competencies was reduced but remained statistically significant. In line with these results, artificial intelligence anxiety and learning engagement played a partial serial mediation role in the relationship between curriculum satisfaction and social–emotional competencies (β = 0.051, SE = 0.010, 95% CI = [0.028, 0.074], *p* < 0.01). The findings may help clarify the importance of curriculum satisfaction, artificial intelligence anxiety, learning engagement, and social–emotional competencies together when designing teacher training curriculum.

## 1 Introduction

The technological revolutions of the first quarter of the 21st century (particularly the rapid integration of generative artificial intelligence/GenAI and large language models/LLMs into educational settings) are associated with a fundamental paradigm shift in teacher education (United Nations Educational, Scientific and Cultural Organization (UNESCO), 2023; Zawacki-Richter et al., 2019). In today’s educational ecosystem, pre-service teachers can no longer be equipped solely with pedagogical and disciplinary knowledge. At the same time, they are expected to possess the psychological and technological resilience to adapt to the digital age (Redecker, 2017). In the new digital ecosystem, teacher education cannot be merely a technical process focused on training pre-service teachers to use technological tools (Darling-Hammond, 2017). Current teacher education policies aim to equip pre-service teachers with a comprehensive set of competencies. These competencies include the ability to collaborate with artificial intelligence (human-AI collaboration), data literacy, and the capacity to manage the ethical, legal, and psychological challenges of the digital world (Hao and Kamil, 2025; Howard et al., 2021). Consequently, modern teacher education should go beyond technical skills (hard skills). It should also include the affective and psychological skills (soft skills) that enable individuals to thrive in the digital world (Malynovska and Barantsova, 2024). Traditional teacher education has historically focused on teachers as “knowledge transmitters” who convey information, maintain classroom order, and implement standard curriculum content (Darling-Hammond, 2017). However, as artificial intelligence has gained new features (such as personalized learning pathways, automated assessment, lesson planning, and real-time feedback to students), the teacher’s role in the classroom has shifted from the “knowledge source” to “facilitator,” “mentor,” and “emotional guide” (Redecker, 2017).

In higher education and teacher training, the curriculum is the most organized and influential institutional environment in which individuals interact (Barnett and Coate, 2005). Curriculum satisfaction reflects pre-service teachers’ perceptions of the curriculum. It goes beyond a mere sense of satisfaction and represents an important environmental input associated with their professional development (Fang et al., 2024). In the educational literature, curriculum satisfaction is defined as a cognitive and affective assessment of the curriculum’s effectiveness in meeting students’ academic expectations, professional needs, and goals (Pucel and Stertz, 2005). For pre-service teachers, curriculum satisfaction is closely related to “cognitive readiness,” in terms of how well the curriculum has prepared them for real classroom settings and future professional demands (Al-Thani et al., 2026). In higher education, this concept encompasses not only satisfaction with course content but also a multidimensional framework that includes the curriculum’s relevance, pedagogical quality, balance between theory and practice, and technological infrastructure (Bahat and Karakütük, 2023). Pre-service teachers’ curriculum satisfaction reflects how well the curriculum integrates disruptive technologies (such as artificial intelligence, generative AI, and smart data analytics) into the pedagogical process (Scherer and Teo, 2019). A high-quality teacher training curriculum should build a strong bridge between theoretical foundations and practical processes, such as school experience and teaching practice (Darling-Hammond, 2017). When pre-service teachers have opportunities to apply theoretical knowledge through digital tools and course activities, they may report higher levels of curriculum satisfaction (Scherer and Teo, 2019). Higher curriculum satisfaction is linked to professional knowledge, pedagogical skills, and supportive learning environments. In supportive learning environments, pre-service teachers can develop a stronger sense of professional identity and social–emotional competencies, such as self-awareness, self-management, interpersonal skills, and responsible decision-making (Spilt et al., 2011; Wu and Qin, 2025). Based on these explanations, Hypothesis 1. (H1) was proposed in this study.

*H1: Pre-service teachers’ curriculum satisfaction (CS) is associated with social–emotional competencies (SEC) positively and significantly.*

For pre-service teachers, the curriculum is a key environmental factor that shapes professional development and adaptation to technology. Individuals with higher levels of curriculum satisfaction tend to view artificial intelligence as a supportive educational resource rather than a potential threat (Beirat et al., 2025). As generative artificial intelligence tools rapidly spread into educational settings, “artificial intelligence (AI) anxiety” has emerged as a new area of research. AI anxiety refers to feelings of professional insecurity, loss of role, and future threats (Wang and Wang, 2022). Accordingly, pre-service teachers with higher curriculum satisfaction may perceive artificial intelligence more positively and report lower levels of AI anxiety (Luckin et al., 2016; Zawacki-Richter et al., 2019). Based on these explanations, this study proposed Hypothesis 2 (H2).

*H2: Pre-service teachers’ curriculum satisfaction (CS) is associated with artificial intelligence anxiety (AIA) negatively and significantly.*

Artificial intelligence (AI) anxiety may consume cognitive and psychological resources. AI anxiety may be linked to lower academic focus and participation (Lopez-Garcia et al., 2022). Concerns about the potential impact of artificial intelligence on professional futures may be associated with cognitive burden and educational difficulty (course content, academic tasks, and learning activities) (Howard et al., 2021). Learning engagement is an important component of the educational process because it reflects behavioral, cognitive, and affective participation in academic activities. In contemporary teacher education, learning engagement is particularly relevant because pre-service teachers are expected to participate actively in courses, collaborative activities, and digital learning environments. The literature indicates that technological and situational anxieties may be associated with lower academic focus (Lopez-Garcia et al., 2022). In this context, AI anxiety may be an important affective concern in educational settings because of the widespread adoption of AI technologies. Pre-service teachers experiencing higher levels of AI anxiety may report greater uncertainty about their professional futures and lower confidence in digital competencies (Cantaş et al., 2026). Previous research suggests that lower perceived control over technological change may be associated with greater AI anxiety and lower motivation, cognitive effort, and learning engagement (Niu et al., 2026). Similarly, pre-service teachers who perceive artificial intelligence as a professional threat may demonstrate lower participation in learning environments, online activities, and technology-supported academic processes (Ayanwale et al., 2025). Empirical findings indicate that individuals experiencing higher levels of AI anxiety may participate less frequently in course discussions, collaborative learning processes, and other academic activities (Rocca, 2010). Conversely, pre-service teachers with lower AI anxiety may report higher levels of cognitive, affective, and behavioral engagement in courses, projects, and learning environments (Hündür et al., 2026). Based on these explanations, this study proposed Hypothesis 3 (H3).

*H3: Pre-service teachers’ artificial intelligence anxiety (AIA) is associated with learning engagement (LE) negatively and significantly.*

As the role of artificial intelligence in education continues to expand, social–emotional competencies (SEC), including interpersonal relationships, empathy, and emotional intelligence, are becoming increasingly important in teacher education. These competencies are essential to the teaching profession (Jennings and Greenberg, 2009). Teacher training curricula should give pre-service teachers opportunities to develop emotional stability, social competencies, and effective leadership skills. Through active participation in course activities, peer groups, online learning environments, collaborative projects, and academic discussions, pre-service teachers can develop social–emotional competencies such as self-awareness, self-management, social awareness, relationship skills, and responsible decision-making (Collaborative for Academic, Social, and Emotional Learning (CASEL), 2020; Jennings and Greenberg, 2009).

The social–emotional learning (SEL) framework (Collaborative for Academic, Social, and Emotional Learning (CASEL), 2020) helps explain the association between learning engagement and social–emotional competencies. Active participation in course activities, peer groups, online learning environments, collaborative projects, and academic discussions gives pre-service teachers opportunities to practice social–emotional competencies such as interpersonal skills, self-management, and responsible decision-making (Collaborative for Academic, Social, and Emotional Learning (CASEL), 2020; Spilt et al., 2011). Social–emotional competencies are recognized as a core outcome of teacher education because the teaching profession requires significant emotional labor, classroom management, and interpersonal interaction (Jennings and Greenberg, 2009). Empirical findings also indicate a positive relationship between learning engagement and dimensions of social–emotional competencies such as self-awareness, interpersonal relationships, collective relationships, and responsible decision-making (Chiara, 2020). Therefore, learning engagement may represent an important experiential context for pre-service teachers’ social–emotional competencies (Jennings and Greenberg, 2009; Mujallid, 2024). Based on these explanations, this study proposed Hypothesis 4 (H4).

*H4: Pre-service teachers’ learning engagement (LE) is associated with social–emotional competencies (SEC) positively and significantly.*

Within the input–output-process (IPO) framework, curriculum satisfaction represents an institutional input that may relate to psychological processes. Accordingly, curriculum satisfaction may be associated with lower AI anxiety. AI anxiety may be linked to social–emotional competencies, such as emotional regulation and psychological well-being (Li Q. et al., 2026). Therefore, AI anxiety may represent a psychological process in the relationship between curriculum satisfaction and social–emotional competencies. Conversely, pre-service teachers satisfied with curriculum content, instructional quality, and academic support may report higher levels of cognitive, affective, and behavioral engagement in courses, project work, and other academic activities (Li H. et al., 2026; Soliba et al., 2025). Higher learning engagement provides opportunities for communication with peers, collaboration, and emotional regulation. These experiences are relevant to self-awareness, self-management, relationship skills, social awareness, and responsible decision-making (Collaborative for Academic, Social, and Emotional Learning (CASEL), 2020). Therefore, learning engagement may represent a behavioral process in the relationship between curriculum satisfaction and social–emotional competencies. Based on these explanations, this study proposed Hypotheses 5 and 6 (H5 and H6).

*H5: Artificial intelligence anxiety (AIA) plays a mediation role in the relationship between pre-service teachers’ curriculum satisfaction (CS) and social–emotional competencies (SEC).*

*H6: Learning engagement (LE) plays a mediation role in the relationship between pre-service teachers’ curriculum satisfaction (CS) and social–emotional competencies (SEC).*

Finally, the serial mediation model provides a framework for understanding pre-service teachers’ professional development in the digital age (Wang et al., 2024). In this context, the proposed serial mediation model examines the following pathway: Curriculum Satisfaction → Artificial Intelligence Anxiety → Learning Engagement → Social–Emotional Competencies. In this proposed pathway, curriculum satisfaction is associated with AI anxiety, AI anxiety with learning engagement, and learning engagement with social–emotional competencies. This pathway tests whether curriculum satisfaction is associated with social–emotional competencies through the serial mediation roles of AI anxiety and learning engagement. Based on these explanations, this study proposed Hypothesis 7 (H7).

*H7: Artificial intelligence anxiety (AIA) and learning engagement (LE) play a serial mediation role in the relationship between pre-service teachers’ curriculum satisfaction (CS) and social–emotional competencies (SEC).*

To provide a coherent and integrated understanding of structural relationships, this study is grounded in the input-process-output (IPO) framework (Bushnell, 1990). Structural relationships in higher education are too complex to capture through isolated linear pathways. Therefore, the IPO framework allows us to map whether curriculum satisfaction is associated with social–emotional competencies through the serial mediation roles of AI anxiety and learning engagement. Within this framework, curriculum satisfaction represents an input, AI anxiety represents an affective process, and learning engagement represents a behavioral process. Social–emotional competencies represent a developmental outcome. Although curriculum satisfaction (Fang et al., 2024), artificial intelligence anxiety (Beirat et al., 2025), learning engagement (Shao et al., 2025), and social–emotional competencies (Jennings and Greenberg, 2009) have been examined independently in the literature, studies investigating how these variables are connected within a sequential pathway remain limited. Existing studies generally examine these variables separately or within simple binary relationships (Fang et al., 2024; Jennings and Greenberg, 2009; Scherer and Teo, 2019). This study addresses important theoretical, empirical, and contextual gaps in the literature by examining the relationships among curriculum satisfaction, artificial intelligence anxiety, learning engagement, and social–emotional competencies in teacher education. The Turkish higher education system also offers an important opportunity to test a serial mediation model that examines the structural relationships among pre-service teachers’ curriculum satisfaction, artificial intelligence anxiety, learning engagement, and social–emotional competencies. Historically, the Turkish teacher education context, characterized by a centralized structure, has undergone a paradigm shift as the Council of Higher Education delegated curriculum-setting authority to universities. Over time, the system has placed greater emphasis on institutional responsibility for curriculum design, implementation, and quality assurance. In this changing context, external accreditation has become an important mechanism for evaluating teacher education quality. This accreditation provides an externally evaluated institutional context in which pre-service teachers experience their courses and curriculum (Council of Higher Education (CoHE), 2020). In this regard, curriculum satisfaction is a critical measure of pre-service teachers’ satisfaction with the curriculum developed by the university. Furthermore, AI anxiety, which this study addresses, is closely linked to fears of job displacement and losing one’s position to robots or algorithms in the future (Wang and Wang, 2022). Similarly, the 2026–2030 National Artificial Intelligence Action Plan, which aims to analyze how artificial intelligence would replace professions, raises concerns about the future of the teaching profession (Ministry of Industry and Technology, 2026). To address these concerns, the Council of Higher Education (CoHE) has published ethical and human-centered guidelines emphasizing that technology cannot replace human presence (Council of Higher Education (CoHE), 2024). Within Türkiye’s educational system, teaching is a profession that requires a high level of emotional labor. Therefore, pre-service teachers should develop the social–emotional competencies that make the profession unique and irreplaceable.

Although AI anxiety has emerged as an important research topic with the rapid integration of generative AI into educational settings, previous research has largely focused on scale development and descriptive statistics (Goyal, 2025). Rapid advances in AI technologies are raising uncertainty about professional roles and future teaching practices. However, fewer studies have examined how AI anxiety relates to pre-service teachers’ academic practices and learning engagement (Wu and Li, 2025). At the same time, the literature has tended to treat social–emotional competencies separately from curriculum experiences and technological challenges in education (Tondeur et al., 2019). The present study addresses this gap by bringing these four constructs together within a two-mediator serial mediation model: Curriculum Satisfaction → AI Anxiety → Learning Engagement → Social–Emotional Competencies. Rather than examining simple binary relationships, this study provides an integrated framework. This structure offers a more coherent theoretical perspective on how curriculum satisfaction, artificial intelligence anxiety, learning engagement, and social–emotional competencies may be interconnected in teacher education. This study also has practical significance for teacher education institutions, curriculum designers, and education policymakers in the age of AI. The findings may clarify the importance of curriculum satisfaction, AI anxiety, learning engagement, and social–emotional competencies together when designing teacher training curricula. Accordingly, this study primarily clarifies how these variables relate within a sequential pathway. In this context, the following hypotheses have been proposed.

*H1*: Pre-service teachers’ curriculum satisfaction (CS) is associated with social–emotional competencies (SEC) positively and significantly.

*H2*: Pre-service teachers’ curriculum satisfaction (CS) is associated with artificial intelligence anxiety (AIA) negatively and significantly.

*H3*: Pre-service teachers’ artificial intelligence anxiety (AIA) is associated with learning engagement (LE) negatively and significantly.

*H4*: Pre-service teachers’ learning engagement (LE) is associated with social–emotional competencies (SEC) positively and significantly.

*H5*: Artificial intelligence anxiety (AIA) mediates the relationship between pre-service teachers’ curriculum satisfaction (CS) and social–emotional competencies (SEC).

*H6*: Learning engagement (LE) mediates the relationship between pre-service teachers’ curriculum satisfaction (CS) and social–emotional competencies (SEC).

*H7*: Artificial intelligence anxiety (AIA) and learning engagement (LE) play a serial mediation role in the relationship between pre-service teachers’ curriculum satisfaction (CS) and social–emotional competencies (SEC).

## 2 Method

### 2.1 Research model

This study examined the conceptual relationships among “curriculum satisfaction,” “AI anxiety,” “learning engagement,” and “social–emotional competencies” using a correlational survey model. The correlational survey model enables testing complex, multi-variable relationships and examining both direct and indirect relationships among variables (Creswell, 2012; Fraenkel and Wallen, 2009). In this study, a correlational survey model was used to examine the serial mediation role of artificial intelligence anxiety and learning engagement in the relationship between pre-service teachers’ curriculum satisfaction and social–emotional competencies, using Model 6 in PROCESS Macro Ver. 4.1 (see Figure 1).

**Figure 1:**
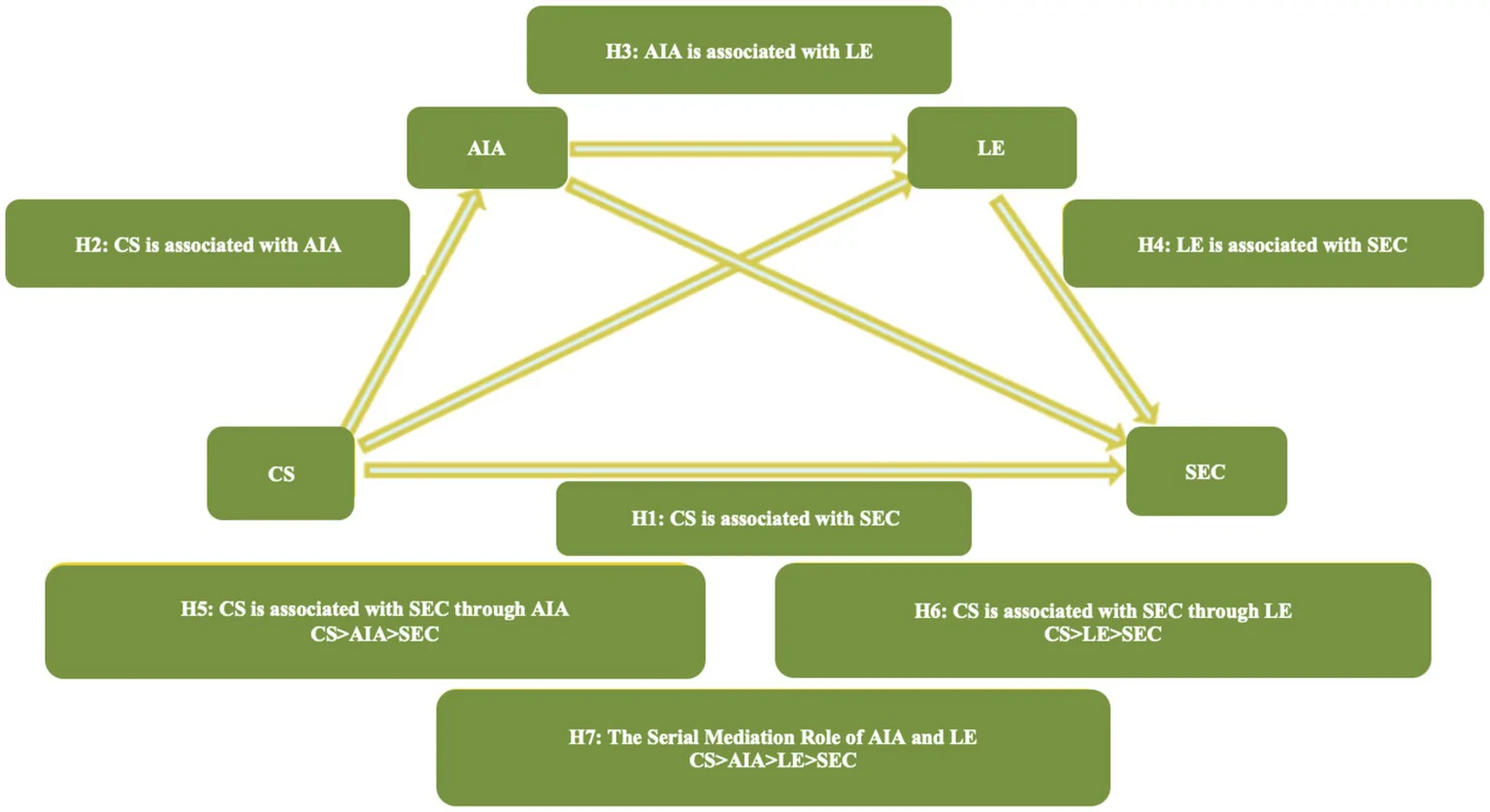
Proposed research model and hypotheses.

### 2.2 Research group

A convenience sampling method was used to select the pre-service teachers who participated in the study. Using this method, the researcher included individuals who were easily accessible in terms of time, money, effort, and other factors (Büyüköztürk et al., 2016). All study procedures were conducted in accordance with the institutional review board’s ethical guidelines and the Declaration of Helsinki. The survey form was administered online via Google Forms. The researcher initially sent invitations to participants. Each invitation included a brief introduction to the study’s purpose and a link to the survey form. All participants were informed about the study’s purpose, procedures, and their right to withdraw at any stage of the study. The researcher obtained written informed consent from all participants.

The study’s target population comprised pre-service teachers enrolled in undergraduate teacher education programs in Türkiye. The accessible population consisted of approximately 850 pre-service teachers enrolled in the faculty of education at a public university in Türkiye. The researcher used a convenience sampling method to recruit participants from the accessible population. Of the approximately 850 pre-service teachers in the accessible population, 700 were invited to participate in the study. Among those invited, 590 returned the questionnaire, yielding an initial response rate of 84.3%. During data screening, 40 returned questionnaires were excluded because they were incomplete or did not provide usable responses for analysis. The remaining 550 questionnaires formed the final analytic sample, corresponding to a final usable-response rate of 78.6% of the invited participants. Consequently, this study included 550 pre-service teachers from a public university in Türkiye, shaped by the Council of Higher Education’s reform delegating authority over the teacher training curriculum and national artificial intelligence initiatives. The research group included 65.8% (*f* = 362) female pre-service teachers and 34.2% (*f* = 188) male pre-service teachers. By grade level, fourth-year students constituted the largest group at 37.3%, followed by third-year (26.9%), second-year (20.4%), and first-year students (15.5%). By department, 29.8% of participants were enrolled in the early childhood and primary education program, 22.2% in the guidance and psychological counseling program, 19.6% in the Turkish and social sciences program, 16.5% in the mathematics and science program, and 11.8% in the music education program.

### 2.3 Data collection tools

The data collection tools were described below.

#### 2.3.1 Student satisfaction scale (EF-SSS)

The Student Satisfaction Scale (EF-SSS), developed by Şahin (2009), was used to determine participants’ levels of curriculum satisfaction. The scale consists of 40 items and 6 sub-dimensions. The sub-dimensions are faculty members, advising, administration, resources, computer availability, and courses and curriculum. The scale uses a five-point Likert-type scale, and items are rated from 1= “I am not at all satisfied” to 5= “I am completely satisfied.” In the scale development study, Cronbach’s alpha internal consistency coefficients for the subscales ranged from 0.68 to 0.91 (Şahin, 2009).

In the present study, the courses and curriculum sub-dimension was used because it best reflected the conceptualization of curriculum satisfaction adopted in this study. The courses and curriculum sub-dimension consisted of items assessing participants’ satisfaction with the courses and curriculum. The other sub-dimensions assessed broader aspects of the university experience. Therefore, these dimensions were not included in the measurement of curriculum satisfaction in this study. In this study, model fit indices were within the acceptable range (ℵ^2^/df = 1.81, RMSEA = 0.032, CFI = 0.94, TLI = 0.93), and standardized factor loadings ranged from 0.577 to 0.836. Cronbach’s alpha internal consistency coefficient was 0.908, and item-total test correlation values ranged from 0.567 to 0.746. These results demonstrated that the scale was a valid and reliable measurement tool.

#### 2.3.2 Artificial intelligence anxiety (AIA) scale

The Artificial Intelligence Anxiety (AIA) Scale, adapted into Turkish by Akkaya et al. (2021), was used in this study to determine participants’ levels of artificial intelligence anxiety. The original scale was developed by Wang and Wang (2022). Cronbach’s alpha for the original scale was 0.964. The reliability coefficients for the four factors were as follows: Learning = 0.974, job replacement = 0.917, sociotechnical blindness = 0.917, and artificial intelligence configuration = 0.961. These values indicated acceptable internal consistency. In conclusion, the 21-item scale was reliable. However, future studies should apply confirmatory factor analysis (CFA) (Wang and Wang, 2022).

The Turkish version of the scale consists of 16 items and 4 sub-dimensions. The sub-dimensions are learning, job replacement, sociotechnical blindness, and artificial intelligence configuration. The scale is a five-point Likert-type scale, and participants rate each item from 1 = Strongly Disagree to 5 = Strongly Agree. In the Turkish adaptation study, the scale’s four-factor structure was confirmed. Confirmatory factor analysis indicated that the model fit indices were within an acceptable range. Additionally, the scale’s overall internal consistency coefficient was Cronbach’s α = 0.937. Cronbach’s α coefficients for the sub-scales were reported as 0.948 for learning, 0.895 for job replacement, 0.875 for sociotechnical blindness, and 0.950 for artificial intelligence configuration (Akkaya et al., 2021).

In this study, the standardized factor loadings ranged from 0.571 to 0.813. In addition, the scale’s ℵ^2^/df (4.71) was less than 5. Moreover, CFI (0.92), TLI (0.94), RMSEA (0.075), and SRMR (<0.08) fell within acceptable ranges, indicating good validity. Cronbach’s alpha was 0.903. These findings indicated that the Turkish version of the scale was a valid and reliable measurement tool.

#### 2.3.3 Student engagement scale

The Student Engagement Scale, developed by Özer and Atik (2014), was used to determine participants’ levels of learning engagement. The scale consists of 11 items and exhibits a single-factor structure. The five-point Likert- type scale ranges from “Never” to “Always.” Higher scores indicate higher levels of student engagement. Exploratory factor analysis showed a single-factor structure that explained 43.51% of the total variance. Factor loadings ranged from 0.58 to 0.72. Cronbach’s alpha internal consistency coefficient was 0.87. CFA results were as follows: (ℵ^2^ = 153.77, df = 39, ℵ^2^/df = 3.94, GFI = 0.97, AGFI = 0.94, NNFI = 0.98, CFI = 0.98, RMSEA = 0.046, RMR = 0.039, SRMR = 0.034 (Özer and Atik, 2014).

In the present study, model fit indices were within the acceptable range (ℵ^2^/df = 1.86, RMSEA = 0.034, CFI = 0.93, TLI = 0.95), and standardized factor loadings ranged from 0.583 to 0.805. Cronbach’s alpha internal consistency coefficient was 0.906, and item-total test correlation values ranged from 0.556 to 0.733. These findings indicated that the scale was a valid and reliable measurement tool.

#### 2.3.4 Social–emotional expertise scale

The Social–Emotional Expertise Scale, adapted into Turkish by Ay and Temel (2021), was used in this study. The original scale was developed by McBrien et al. (2020). For construct validation, three experiments were conducted to develop a self-report measure. The scale consists of 25 items, each scored on a five-point Likert-type scale. Experiment 1 tested 76 items to measure social–emotional expertise. The resulting 25-item scale was reliable. Test–retest: r(80) = 0.82, *p* < 0.001, and internally consistent (Cronbach’s α = 0.90). Experiments 2 and 3 examined the relationships between social–emotional expertise and related constructs. Convergent constructs, such as emotional intelligence, r(885) = 0.62, *p* < 0.01, and social anxiety, r(885) = −0.59, *p* < 0.01, and discriminant constructs, such as social desirability, r(885) = 0.19, *p* < 0.01, and self-monitoring, r(885) = 0.28, *p* < 0.01, were related in the expected directions. Additionally, two factors were identified: Adaptability and expressivity. These factors correlated with related constructs (McBrien et al., 2020).

The Turkish version of the scale consists of 25 items, each scored on a five-point Likert-type scale. The scale contains no reverse-scored items. The scale comprises two sub-scales. (i) Adaptability: This sub-scale measures the ability to adapt easily to various social and emotional interpersonal situations. Sample item: When someone I know is sad, I know what the right thing is to say and do. (ii) Expressiveness: The ability to express emotions to others. Sample item: I am animated and lively when I speak. Cronbach’s alpha was 0.804 for adaptability and 0.736 for expressiveness. Finally, Cronbach’s alpha was 0.855 for the whole scale. The results of the CFA showed that the model fit indices were acceptable (RMSEA = 0.059, GFI = 0.90, AGFI = 0.90, CFI = 0.94, IFI = 0.94, NNFI = 0.94). Based on the confirmatory factor analysis, it was concluded that the two-factor structure of the scale has been validated in the Turkish cultural context (Ay and Temel, 2021).

In this study, model fit indices fell within the acceptable range (ℵ^2^/df = 1.85, RMSEA = 0.036, CFI = 0.96, TLI = 0.95), and standardized factor loadings ranged from 0.575 to 0.821. Cronbach’s alpha internal consistency coefficient was 0.911, and item-total test correlation values ranged from 0.558 to 0.725. These results indicated that the scale was a valid and reliable measurement tool.

### 2.4 Data analysis

The data analysis phase is explained in this section.

In the data analysis phase, four fundamental assumptions were initially checked: Normality (univariate and multivariate), linearity, equivariance, and multicollinearity (Kline, 2011).

Table 1 provides information regarding the assumptions.

**Table 1:** Assumptions regarding the analyses.

Variables	Univariate normality	Multivariate normality and linearity	Multicollinearity	Equivariance
All Variables	For CS; SV: −0.887 KV: 0.625 For AIA; SV: −0.762 KV: −0.078 For LE; SV: 1.017 KV: −0.118 For SEC; SV: −0.492 KV: 0.017	Scattering Matrix = Close to Ellipse Form	Correlation coefficient between independent variables= It ranged from 0.41 to 0.54.	Box M Test = *p* > 0.05

After examining the dataset against four basic assumptions, the data analysis proceeded. In the data analysis process, descriptive statistics and correlation analysis were used. The proposed model was analyzed using SPSS 24 software via the PROCESS macro. In addition, a two-mediator serial mediation analysis was used to determine the direct and indirect relationships among the research variables. The two-mediator serial mediation model included the pathway (Curriculum Satisfaction → AI Anxiety → Learning Engagement → Social–Emotional Competencies). In this model, the sequential relationships among the study variables are determined, unlike the parallel mediation model, which assumes the mediator variables are unrelated (Vartanian et al., 2016). In this study, the researcher used the IBM SPSS PROCESS macro (Model 6-Two-Mediator Serial Mediation Analysis/Model) based on the bootstrap method developed by Andrew F. Hayes. The PROCESS macro is considered useful for estimating direct and indirect relationships in a two-mediator serial mediation model (Hayes, 2013). Therefore, this study used the PROCESS macro (Model 6) to obtain the findings.

Figure 1 shows the proposed research model and hypotheses.

In line with Model 6, the indirect relationships of each mediator variable could be examined separately [AI anxiety (Hypothesis 5) and learning engagement (Hypothesis 6)]. The serial mediation role of the two variables [AI anxiety and learning engagement (Hypothesis 7)] could also be examined (Hayes, 2013). In this study, the variables were as follows: Curriculum satisfaction = exogenous variable; social–emotional competencies = endogenous variable; AI anxiety = first mediator variable; and learning engagement = second mediator variable.

## 3 Results

### 3.1 Findings regarding the descriptive statistics and correlation analysis

The findings obtained from descriptive statistics and correlation analysis are presented in this section.

Table 2 presents descriptive statistics and correlation values for the research variables.

**Table 2:** Descriptive statistics and correlation values related to research variables.

Variables	$\bar{X}$	SD	CS	AIA	LE	SEC
CS	3.82	0.68	1	−0.45*	0.54*	0.49*
AIA	51.20	9.85		1	−0.41*	−0.36*
LE	122.40	19.10			1	0.58*
SEC	78.50	12.90				1

*p < 0.05.

As shown in Table 2, Pearson correlation analysis was conducted because the variables were normally distributed, and it was concluded that all variables were significantly related to each other (*p* < 0.05). The results of the correlation analyses showed that curriculum satisfaction was negatively correlated with artificial intelligence anxiety (AIA) (r = −0.45, *p* < 0.01). Curriculum satisfaction was positively associated with learning engagement (r = 0.54, *p* < 0.01) and social–emotional competencies (r = 0.49, *p* < 0.01). Additionally, a negative and significant relationship was found between AI anxiety and learning engagement (r = −0.41, *p* < 0.01). There was a significant negative relationship between AI anxiety and social–emotional competencies (r = −0.36, *p* < 0.01). There was a significant positive relationship between learning engagement and social–emotional competencies (r = 0.58, *p* < 0.01).

### 3.2 Findings regarding the serial mediation analysis with two mediator variables

Findings from the serial mediation analysis are presented in this section.

Table 3 presents the findings regarding the hypotheses within the scope of the two-mediator serial mediation analysis.

**Table 3:** Hypotheses within the scope of the two-mediator serial mediation analysis.

Hypotheses	Bootstrap coefficient^a^	Standard error	*p*	Lower limit-Upper limit^b^	Acceptance of the hypothesis
H1: CS → SEC	0.240**	0.042	*p* < 0.01	0.148 to 0.332	ACCEPTANCE
H2: CS → AIA	−0.450**	0.038	*p* < 0.01	−0.525 to −0.375	ACCEPTANCE
H3: AIA → LE	−0.230**	0.041	*p* < 0.01	−0.312 to −0.148	ACCEPTANCE
H4: LE → SEC	0.410**	0.045	*p* < 0.01	0.321 to 0.499	ACCEPTANCE
H5: CS → AIA → SEC	0.063**	0.016	*p* < 0.01	0.024 to 0.098	ACCEPTANCE
H6: CS → LE → SEC	0.138**	0.021	*p* < 0.01	0.095 to 0.181	ACCEPTANCE
H7: CS → AIA → LE → SEC	0.051**	0.010	*p* < 0.01	0.028 to 0.074	ACCEPTANCE

a: 5.000 bootstrap sample, b: 95% confidence interval. Total Impact: 0.492; Direct Impact: 0.240; Total Indirect Impact: 0.252. **p < 0.01.

As shown in Table 3, curriculum satisfaction was positively associated with social–emotional competencies (β = 0.240, SE = 0.042, 95% CI = [0.148, 0.332], *p* < 0.01). In addition, curriculum satisfaction was negatively associated with AI anxiety (β = −0.450, SE = 0.038, 95% CI = [−0.525, −0.375], *p* < 0.01). AI anxiety was also negatively associated with learning engagement (β = −0.230, SE = 0.041, 95% CI = [−0.312, −0.148], *p* < 0.01). Furthermore, learning engagement was positively associated with social–emotional competencies (β = 0.410, SE = 0.045, 95% CI = [0.321, 0.499], *p* < 0.01). Finally, these direct relationships were significant because the confidence intervals did not include zero. Based on these results, H1, H2, H3, and H4 were accepted.

As for the indirect relationships, curriculum satisfaction was associated with social–emotional competencies through AI anxiety (β = 0.063, SE = 0.016, 95% CI = [0.024, 0.098], *p* < 0.01). Furthermore, curriculum satisfaction was associated with social–emotional competencies through learning engagement (β = 0.138, SE = 0.021, 95% CI = [0.095, 0.181], *p* < 0.01). Finally, these indirect relationships were significant because the confidence intervals did not include zero. Based on these results, H5 and H6 were also accepted.

In addition, AI anxiety and learning engagement played a serial mediation role in the relationship between curriculum satisfaction and social–emotional competencies (β = 0.051, SE = 0.010, 95% CI = [0.028, 0.074], *p* < 0.01). The indirect relationship was significant because the confidence interval did not include zero. Moreover, curriculum satisfaction was associated with social–emotional competencies (β = 0.492, *p* < 0.01). When the model included AI anxiety and learning engagement simultaneously, the relationship between curriculum satisfaction and social–emotional competencies was reduced but remained statistically significant (β = 0.240, *p* < 0.01). Thus, AI anxiety and learning engagement played a partial serial mediation role in the relationship between curriculum satisfaction and social–emotional competencies. Based on these results, H7 was accepted.

## 4 Discussion

The present study examined the relationships among pre-service teachers’ curriculum satisfaction (CS), artificial intelligence anxiety (AIA), learning engagement (LE), and social–emotional competencies (SEC) using a serial mediation model. The interpretation of these relationships was primarily grounded in the input-process-output (IPO) framework (Bushnell, 1990). In this framework, curriculum satisfaction served as the input, artificial intelligence anxiety and learning engagement as affective and behavioral processes, and social–emotional competencies as the developmental output. The social–emotional learning (SEL) framework (Collaborative for Academic, Social, and Emotional Learning (CASEL), 2020) was incorporated as a complementary perspective to clarify specific relationships within this structure.

The positive relationship between curriculum satisfaction and social–emotional competencies (β = 0.240, SE = 0.042, 95% CI [0.148, 0.332], *p* < 0.01; r = 0.49, *p* < 0.01) was broadly consistent with prior study results. For example, Wang et al. (2025) reported positive associations between curriculum satisfaction (such as active learning, teaching, and clarity of goals and standards) and students’ social–emotional competencies. This study suggested that perceptions of curriculum satisfaction were relevant to socio-emotional development across higher education contexts. However, the present study differed from previous studies. The present study located curriculum satisfaction at the input stage of the IPO framework. Jennings and Greenberg (2009) provided complementary conceptual support by emphasizing the importance of teachers’ social–emotional competencies for supportive relationships, classroom functioning, and social–emotional learning. Thus, this relationship was consistent with earlier evidence. However, this study extended the discussion from specific teaching practices toward pre-service teachers’ perceptions of the curriculum. In teacher education, these perceptions may matter because the curriculum provides the context in which future teachers develop both professional and social–emotional competencies (Wang et al., 2025). Jennings and Greenberg (2009) also emphasized the importance of social–emotional competencies for teacher development, supportive relationships, and classroom functioning. Within the IPO framework, curriculum satisfaction can be viewed as an input that is statistically associated with the developmental output represented by social–emotional competencies.

Another finding was a negative relationship between curriculum satisfaction and artificial intelligence anxiety (β = −0.450, SE = 0.038, 95% CI [−0.525, −0.375], *p* < 0.01; r = −0.45, *p* < 0.01). Although emerging literature focuses on the relationships among educational support, technological competencies, and AI-related anxiety, these variables have not been linked to curriculum satisfaction. Chai and Sha (2025) reported significant relationships among educational support, AI anxiety, and AI self-efficacy. Furthermore, Cengiz and Peker (2025) identified significant relationships among university students’ generative AI acceptance, AI anxiety, attitudes toward AI, and AI literacy. These studies highlighted the relevance of educational support, technological competence, and AI-related anxiety. Therefore, the present study extends previous research by positioning curriculum satisfaction as an input associated with AI anxiety. This finding is particularly relevant to teacher education. Pre-service teachers are preparing to work in classrooms where AI and other digital technologies continue to expand. Therefore, their preparation involves more than technical knowledge. It also includes readiness, the ability to respond to technological uncertainty, digital pedagogical knowledge, and confidence in using AI-related tools (Ayanwale et al., 2025).

The negative relationship between artificial intelligence anxiety and learning engagement (β = −0.230, SE = 0.041, 95% CI [−0.312, −0.148], *p* < 0.01; r = −0.41, *p* < 0.01) aligns with recent empirical research examining AI anxiety and learning engagement. Ding and Xue (2025) reported a negative relationship between AI anxiety and learning engagement. AI anxiety is an important affective component in learning contexts. Ayanwale et al. (2025) examined AI anxiety together with readiness, confidence, attitudes, and learning engagement. These studies provide evidence that AI anxiety is related to students’ learning engagement. This relationship has practical relevance for teacher education. Pre-service teachers need to engage actively in coursework, teaching practice, collaborative tasks, and professional learning. AI anxiety may be associated with participation in learning activities. Such activities may provide a supportive context for addressing artificial intelligence anxiety while maintaining learning engagement (Ayduğ and Altınpulluk, 2025).

The positive relationship between learning engagement and social–emotional competencies (β = 0.410, SE = 0.045, 95% CI [0.321, 0.499], *p* < 0.01; r = 0.58, *p* < 0.01) aligns with recent empirical evidence involving pre-service teachers. Li et al. (2026) reported significant relationships between dimensions of social–emotional competencies and learning engagement. Similarly, Mujallid (2024) examined digital active learning, student engagement, and social–emotional learning skills in higher education. This study highlighted the relationship between course design, learning activities, learning engagement, and social–emotional learning. These studies provide empirical support for the relationship between learning engagement and social–emotional development in educational settings. The present study extends the literature by linking learning engagement to the social–emotional competencies within the IPO framework. The relationship is also conceptually consistent with the social–emotional learning (SEL) framework (CASEL, 2020), which emphasizes opportunities for interaction, collaboration, self-management, social awareness, relationship skills, and responsible decision-making. Jennings and Greenberg (2009) provide theoretical support for the role of social–emotional competencies in teacher education. This relationship is particularly important for teacher education. It is assumed that pre-service teachers cannot develop professional competencies through theoretical knowledge alone. Pre-service teachers also learn through participation in discussions, group activities, teaching practice, peer interaction, and reflection. Such experiences may provide contexts in which social–emotional competencies are practiced (Mwananyama, 2026).

The indirect relationship between curriculum satisfaction and social–emotional competencies through artificial intelligence anxiety (β = 0.063, SE = 0.016, 95% CI [0.024, 0.098], *p* < 0.01) supported the proposed pathway. This indirect relationship was statistically significant because the bootstrap confidence interval did not include zero. As another finding, the indirect relationship between curriculum satisfaction and social–emotional competencies through learning engagement was significant (β = 0.138, SE = 0.021, 95% CI [0.095, 0.181], *p* < 0.01). Finally, curriculum satisfaction was associated with social–emotional competencies through the serial mediation roles of AI anxiety and learning engagement (β = 0.051, SE = 0.010, 95% CI [0.028, 0.074], *p* < 0.01). Moreover, the direct relationship between curriculum satisfaction and social–emotional competencies remained statistically significant after the indirect pathways were included in the model. In line with these results, pre-service teachers satisfied with the current, high-quality curriculum structure may be less likely to perceive artificial intelligence as a major threat to their professional identity and may report lower levels of AI anxiety. Consistent with previous empirical research, pre-service teachers satisfied with curriculum content tend to show higher levels of affective and cognitive engagement in their courses (Zhang et al., 2025). Higher learning engagement may be associated with greater responsibility for learning tasks, more active participation in collaborative learning projects, and stronger involvement in peer communities. These learning experiences may support the development of interpersonal relationships, social awareness, and responsible decision-making skills (Li, 2025; Li H. et al., 2026). Previous empirical studies provide support for different parts of this pathway. Chai and Sha (2025) examined the relationship between educational support and AI anxiety. Ding and Xue (2025) reported a negative relationship between AI anxiety and learning engagement. Ayanwale et al. (2025) examined AI anxiety and engagement-related factors among pre-service teachers. Li H. et al. (2026) reported significant relationships between learning engagement and social–emotional competencies among pre-service teachers. These studies provide important empirical foundations for the relationships examined in the present study. However, these studies differ from the present study in sample and model structure. More importantly, the relevant literature tends to examine curriculum satisfaction, AI anxiety, learning engagement, and social–emotional competencies separately rather than within a single model. Therefore, the present study extends previous studies by integrating these relationships into a serial mediation model. However, this sequence should not be interpreted as evidence of a temporal or causal mechanism because the data were collected using a cross-sectional correlational design.

Taken together, the present study integrates four related constructs into a single model. The results showed that curriculum satisfaction was negatively associated with artificial intelligence anxiety. Artificial intelligence anxiety was negatively associated with learning engagement. Learning engagement was positively associated with social–emotional competencies. Curriculum satisfaction was also positively associated with social–emotional competencies. Previous studies have examined binary relationships among curriculum satisfaction, AI anxiety, learning engagement, and social–emotional competencies. The present study examines these relationships simultaneously within a single model. In summary, these relationships form the proposed pathway of curriculum satisfaction, artificial intelligence anxiety, learning engagement, and social–emotional competencies within an IPO-based serial mediation model. Consequently, this model matters not only for the statistical significance of its pathways but also for integrating these relationships into a coherent theoretical framework.

## 5 Recommendations

Based on the observed associations among the study variables, several recommendations can be offered for pre-service teacher education. First, the negative relationship between curriculum satisfaction and artificial intelligence anxiety suggests that institutions may review the curriculum’s relevance, adequacy, and currency in relation to emerging technologies. When updating teacher training curricula, institutions may consider incorporating relevant content such as artificial intelligence, digital literacy, and the pedagogical use of AI. Such content could provide pre-service teachers with opportunities to develop knowledge and awareness of AI-related issues (Ayduğ and Altınpulluk, 2025; Ayanwale et al., 2025). Second, the negative relationship between artificial intelligence anxiety and learning engagement suggests that designers should consider AI-related concerns when creating learning environments for pre-service teachers. Teacher training curricula may offer pre-service teachers opportunities to discuss their concerns about AI, gain practical experience with AI-supported educational tools, and critically examine the opportunities and challenges of these technologies (Ayanwale et al., 2025; Ding and Xue, 2025). Third, the positive relationship between learning engagement and social–emotional competencies suggests that teacher training curricula may create learning environments that encourage active participation, collaboration, reflection, interaction, and the development of social–emotional competencies (Mwananyama, 2026). Fourth, the positive relationship between curriculum satisfaction and social–emotional competencies suggests that curriculum satisfaction should be considered not only in relation to academic preparation but also to the broader development of pre-service teachers. Therefore, teacher training curricula may include learning experiences that provide opportunities for self-reflection, interpersonal interaction, social awareness, and responsible decision-making alongside academic and technological content (Wang et al., 2025). Finally, the serial mediation role of AI anxiety and learning engagement suggests that these two dimensions may be considered together rather than addressed independently. From an educational perspective, teacher training curricula may develop learning environments in which pre-service teachers can both engage with AI-related technologies and participate actively in meaningful learning activities. For example, AI-supported projects may be combined with collaborative learning, critical reflection, peer discussion, and teaching-practice activities. Such an integrated approach may provide opportunities to address technological concerns while supporting active engagement and broader professional development (Ayanwale et al., 2025). However, all of the recommended practices under this heading should be regarded as possible educational strategies rather than interventions whose effectiveness has been tested in the present study.

## 6 Limitations and future research directions

Several limitations of the present study should be addressed in future research. These limitations also suggest directions for future research. The first limitation is that a cross-sectional design does not allow causal inference among the study variables. Although the theoretical model posits relationships among study variables, the data establish associations at a single point in time. The methodological literature clearly shows that cross-sectional data cannot establish causality or temporal precedence (Maxwell and Cole, 2007). Therefore, future studies should use a longitudinal, experimental, or quasi-experimental design to establish causality between these variables or to understand their temporal ordering. Second, self-reported questionnaires raise the risk of common method bias and other response biases, such as socially desirable responding. Because all measures were self-reported, common method bias was a concern. Established recommendations were followed to minimize common method bias (Podsakoff et al., 2003). Following the guidelines of Podsakoff et al. (2012), several procedures were used to minimize common method bias and increase response accuracy. These included survey instructions, detailed information about the study’s purpose, and assurances of confidentiality and anonymity. The study also clearly stated that participation was voluntary. Participants were not compensated for their involvement in the study. Another limitation is the convenience sampling method. Future studies should use a randomized sampling method and a much larger sample that better represents the general population. The sample’s geographic and institutional homogeneity is another limitation. Therefore, the findings should be interpreted primarily within the Turkish teacher education context and the state university where the research was conducted. Generalizability to other national, educational, and institutional settings should be considered with caution. Future research could replicate the proposed model across different teacher education systems to determine whether the observed relationships remain consistent across diverse educational and cultural contexts. Future research should adopt multi-provincial or cross-national sampling strategies to test the consistency and cross-contextual applicability of the proposed model. Future research could also examine alternative mediator orderings. Additionally, future research could extend the proposed model by incorporating additional psychological, technological, and professional variables relevant to pre-service teacher education. In particular, AI self-efficacy, AI literacy, digital literacy, academic self-efficacy, teacher self-efficacy, professional identity, and perceived institutional support could be examined as potential mediators or moderators.

## Data Availability

The raw data supporting the conclusions of this article will be made available by the authors, without undue reservation.
